# Efficiency of multi-trait, indirect, and trait-assisted genomic selection for improvement of biomass sorghum

**DOI:** 10.1007/s00122-017-3033-y

**Published:** 2017-12-07

**Authors:** Samuel B. Fernandes, Kaio O. G. Dias, Daniel F. Ferreira, Patrick J. Brown

**Affiliations:** 1 Department of Crop Sciences, University of Illinois, 1206 W Gregory Drive, IL Urbana, 61801 USA; 2 Department of Genetics, Luiz de Queiroz College of Agriculture, University of São Paulo, PO Box 83, Piracicaba, SP 13400-970 Brazil; 3 Departamento de Estatística, Universidade Federal de Lavras, 3037, Lavras, MG 37200-000 Brazil

## Abstract

**Key message:**

We compare genomic selection methods that use correlated traits to help predict biomass yield in sorghum, and find that trait-assisted genomic selection performs best.

**Abstract:**

Genomic selection (GS) is usually performed on a single trait, but correlated traits can also help predict a focal trait through indirect or multi-trait GS. In this study, we use a pre-breeding population of biomass sorghum to compare strategies that use correlated traits to improve prediction of biomass yield, the focal trait. Correlated traits include moisture, plant height measured at monthly intervals between planting and harvest, and the area under the growth progress curve. In addition to single- and multi-trait direct and indirect GS, we test a new strategy called trait-assisted GS, in which correlated traits are used along with marker data in the validation population to predict a focal trait. Single-trait GS for biomass yield had a prediction accuracy of 0.40. Indirect GS performed best using area under the growth progress curve to predict biomass yield, with a prediction accuracy of 0.37, and did not differ from indirect multi-trait GS that also used moisture information. Multi-trait GS and single-trait GS yielded similar results, indicating that correlated traits did not improve prediction of biomass yield in a standard GS scenario. However, trait-assisted GS increased prediction accuracy by up to $50\%$ when using plant height in both the training and validation populations to help predict yield in the validation population. Coincidence between selected genotypes in phenotypic and genomic selection was also highest in trait-assisted GS. Overall, these results suggest that trait-assisted GS can be an efficient strategy when correlated traits are obtained earlier or more inexpensively than a focal trait.

**Electronic supplementary material:**

The online version of this article (10.1007/s00122-017-3033-y) contains supplementary material, which is available to authorized users.

## Introduction

Releasing new varieties usually requires evaluation of progenies in a large number of environments. Because the costs of field experiments are becoming the limiting factor (Gawenda et al. 2015; Heslot et al. 2015), strategies that allow rapid, accurate, and resource-efficient predictions are of increasing interest. The application of best linear unbiased prediction (BLUP) using pedigree information Henderson (1975) and more recently using molecular markers (GBLUP) (VanRaden 2008; Hayes et al. 2009b) are examples of efforts to meet those goals.

When GBLUP or other GS models are applied, selection is made on genomic estimated breeding values (GEBVs) calculated from molecular markers and using phenotypic information of a training population. GS has been successfully applied in many animal (Vallée et al. 2014; de los Campos et al. 2013) and plant (Heffner et al. 2011; Heslot et al. 2012) breeding programs, and prediction accuracy (*r*) generally shows a positive correlation with heritability $(h^2)$ (Hayes et al. 2009a). When a focal trait has low $h^2$, indirect or multi-trait GS can be applied to take advantage of correlated traits with higher $h^2$ to increase *r* for the focal trait (Mrode 2014, page 70). Benefits of multi-trait GS over single-trait GS have been reported in simulated (Calus and Veerkamp 2011) and real data (Jia and Jannink 2012; Schulthess et al. 2016).

Sorghum [(*Sorghum bicolor (L.)* Moench] is a multipurpose crop that is grown to produce grain, forage, and most recently biomass for second-generation biofuel production. Some advantages of sorghum as a biomass crop include low implementation cost, short cycle, wide adaptability, mechanized management, and high calorific value in boilers (Vermerris and Saballos 2013; Castro et al. 2015). Biomass yield in sorghum has low heritability (Shiringani and Friedt 2011) and is costly and laborious to phenotype. Correlated traits, including plant height, are much easier and more cost-effective to phenotype and have higher heritability (Monk et al. 1984; Castro et al. 2015; Burks et al. 2015). One previous study applied single-trait GS to predict biomass yield in a diverse photoperiod-sensitive sorghum panel (Yu et al. 2016). Much of the phenotypic variation in biomass yield could be explained in a model including plant height, stalk number, and lodging $(R^2=0.63)$, and indirect GS using these three traits yielded a prediction accuracy only slightly lower than direct GS on biomass yield $(r = 0.71$ versus 0.76). However, the authors did not test multi-trait GS approaches.

In this study, we compare the efficiency of various GS strategies for increasing prediction accuracy of a focal trait, sorghum biomass yield, using information from correlated traits.

## Materials and methods

### Plant material and field experiments

A panel of 453 diverse photoperiod-sensitive sorghum lines was obtained from the United States National Plant Germplasm System (NPGS) and evaluated in Urbana, IL from 2012 to 2014. Along with the diverse panel, the commercial hybrid “Pacesetter” (Richardson Seeds, Vega, TX, USA) was included as check in all years. The experimental design in 2012 was a randomized complete block design with two replications of single row plots with a row length of 7.6 m, 1.5 m alleys and 0.76 m row spacing and a total of 24 rows and 16 columns. Thus, 179 sorghum lines were planted in 2012 and the remaining plots were filled with the commercial hybrid. The experimental design in 2013 and 2014 was an augmented block design with the commercial hybrid included as a check in each block and 24 additional genotypes repeated twice in each year. Each incomplete block consisted of 24 four-row plots with a row length of 3 m, 1.5 m alleys and 0.76 m row spacing and a total of 12 rows and 40 columns. The 480 plots used in 2013 and 2014 were filled with 415 lines, among which 141 lines were also included in 2012. The remaining plots were filled with the check hybrid. The target density in all years was approximately 207, 570 plants/ha, though the final density in 2013 was lower due to climatic conditions and planting error. In each year, field experiments were planted in late May and harvested in early October.

### Phenotyping

Plant height was measured as plot average from the ground to the whorl, at 30 (*H*1), 60 (*H*2), 90 (*H*3) and 120 (*H*4) days after planting. Total plot wet weight (kg) was measured with a forage harvester consisting of a John Deere 5830 tractor with a four-row Kemper head and a weigh wagon modified with load cells accurate to within 1 kg. A 0.5 kg chopped subsample was captured from each plot at harvest, then weighed before and after oven drying at $60~^{\circ }$C for $72'$ to determine moisture content: Moisture (*M*) = (subsample wet weight − subsample dry weight)/subsample wet weight. Biomass yield in dry metric tons per hectare (*Y*) was calculated as: dry metric tons/ha = total plot wet weight $(\mathrm{{kg}}) * (1~-$ plot moisture) / (plot area $(\mathrm{{m}}^2)/10{,}000)$.

### Genotyping

DNA was extracted from dark-grown etiolated seedling tissue in 96-well plates using a CTAB protocol. Illumina libraries were created using two pairs of restriction enzymes: PstI-HF/HinP1I and PstI-HF/BfaI (New England Biolabs, Ipswich, MA). Restriction–ligation was performed in 96-well plates, and unique barcoded adapters were ligated to each DNA sample. 96 DNA samples per library were pooled into a single tube for all subsequent steps including size selection using AMPure beads (Beckman-Coulter, Pasadena, CA, USA), PCR amplification using Phusion polymerase (New England Biolabs), and a second round of a bead-based size selection. Single-end, 100-bp sequencing reads were obtained for all libraries on an Illumina HiSeq2000 instrument following submission protocol to the Keck Center at the University of Illinois. The TASSEL3 GBS pipeline (Glaubitz et al. 2014) was used to identify SNPs, using Bowtie2 (Langmead and Salzberg 2012) for tag alignment. Only reads that perfectly matched a barcode and restriction site overhang were retained. After barcode trimming, a set of “master tags” was generated from the unique 64 bp sequences present at least ten times in the dataset that mapped uniquely to the sorghum genome. SNPs were called by comparing the tags in each individual to the set of master tags at each genomic address. SNPs and individuals with more than $95\%$ missing data as well as SNPs with MAF less than $5\%$ were discarded. Missing data were imputed using BEAGLE4 (Browning and Browning 2011) using a window size and overlap of 500 and 100 SNPs, respectively. The final genotypic dataset consisted of 59264 SNPs with an average MAF of 0.21 and $6.06\%$ heterozygous genotypes.

### Data analysis

Due to the differences in field experimental designs and field heterogeneity across years, as well as for reasons of computational efficiency, a two-stage analysis was performed. In the first stage, a mixed model approach was used to account for spatial variation, generating adjusted means for each genotype in each trial. The most appropriate model for each combination of trait and year was chosen based on the variogram (Gilmour et al. 1997) and the Akaike information criterion (AIC) (Table S1), where the full model is:

1 $$\begin{aligned} y_{ij} = \mu +G_i+B_j+e_{ij}, \end{aligned}$$

Each phenotypic data point ($y_{ij}$) was observed in genotype *i*, block *j*; $\mu$ is a constant; $G_i$ is the fixed effect of the *i*th genotype; $B_j$ is the independent and identically distributed random effect of the *j*th block with $B_j \sim N(0,\sigma _b^2I)$ and $e_{ij}$ is the random effect of residuals, with $\varvec{e}\sim N(0,\sigma ^2_{AR1 \times AR1})$, where $AR(1) \times AR(1)$ is a first-order auto-regressive structure applied to row and column for spatial correction. Adjusted means ($\bar{x}$) were then calculated as the mean of the scaled values from each year.

In the second stage, a GBLUP model was used to obtain genomic predictions for different traits. In addition to predicting each height measurement individually, the area under the growth progress curve (*A*) was also calculated from the adjusted values of all height measurements and analyzed as a different trait. Since all height measurements were 30 days apart, this was obtained from the following simplified equation:

2 $$\begin{aligned} A = \sum _{i=1}^{m} \frac{(h_{i-1}+h_i)}{2}, \end{aligned}$$

where *m* is the number of height measurements, and $h_i$ is height measure at the *i*th observation.

The model used for single-trait GS was:

3 $$\begin{aligned} y_{i} = \mu +g_{i}+e_{i}, \end{aligned}$$

where $y_{i}$ is the adjusted means from the first stage, $\mu$ is a constant; $g_{i}$ is the vector of random effect of genotypes with $\varvec{g}=[g_1, g_2, \cdots , g_n]^\top$ and $\varvec{g}~\sim ~N(0, \varvec{A} \sigma ^2_g )$, where $\sigma ^2_g$ is the additive genetic variance and $\varvec{A}$ is the realized additive relationship matrix calculated from the genotypic dataset using the *A.mat* function from rrBLUP package (Endelman and Jannink 2012); $e_{i}$ is the identical and independently distributed residual with $e_{i}\sim N(0, \sigma ^2_eI)$, where $\sigma ^2_e$ is the residual variance. Genomic heritability ($h_\mathrm{{g}}^2$) was calculated by the ratio of additive and phenotypic variance (de los Campos et al. 2015).

The model used for multi-trait GS with *p* variables, following a notation similar to that used by Ferreira (2011, page 331) was:

4 $$\begin{aligned} \varvec{Y}_{i} = \varvec{\mu }+\varvec{g}_{i}+\varvec{e}_{i}, \end{aligned}$$

where $\varvec{Y}_{i}$ is the vector of multivariate responses associated with genotype *i* $(i=1, 2,\ldots , n)$, in which $\varvec{Y}_{i}=[Y_{i1}, Y_{i2},\ldots ,Y_{ip}]^\top$, $\varvec{\mu }$ is the vector of the constants associated with each trait, with $\varvec{\mu }= [\mu _1, \mu _2,\ldots , \mu _p]^\top$, $\varvec{g}_{i}$ is the vector of random effects of genotype *i* associated with each trait, in which $\varvec{g} = [ \varvec{g}_{1}, \varvec{g}_{2},\ldots , \varvec{g}_{i}, \ldots ,\varvec{g}_{n}]^\top$, $\varvec{g}~\sim ~N_{np}(\varvec{0}, \varvec{G}~\otimes ~\varvec{A} )$, $\varvec{e}_{i}$ is the vector of random effects of residuals from the multivariate model, $\varvec{e} = \left[ \varvec{e}_{1}, \varvec{e}_{2},\ldots ,\varvec{e}_{i}, \ldots ,\varvec{e}_{n}\right] ^\top$, with $\varvec{e}~\sim ~N_{np}(\varvec{0}, \varvec{I}~\otimes ~\varvec{R})$. The matrices **G** and **R** are the variance–covariance matrices (VCOV) for genetic and residual effects, respectively. In both cases, these are assumed to be unstructured, considering correlation for all pairs of traits and specific variances for each trait. The multi-trait model was used in this study for $p=2$. Genetic and residual correlation were obtained from the multi-trait analysis and its respective standard errors were estimated by the Delta method, all of which are given as an output of ASReml-R (Fikret Isik 2017, page 116).

### Cross-validation and prediction accuracy

The prediction accuracy of each model was accessed through $k-fold$ cross-validation with $k=5$, randomly splitting the dataset in five sets and using four of them to predict the remaining set. This process was repeated for each one of the five sets, storing all GEBVs before calculating a single Pearson’s correlation between five folds of GEBVs and adjusted means. This process was repeated 30 times and the same folds were used to perform cross-validation for the different models. Mean and standard deviation of the correlations were calculated and reported as prediction accuracy and its standard deviation, respectively. Training set and validation set varied according to the model used (Table 1).

**Table 1:** Training and validation sets used in cross-validation for each genomic selection model

	Model	Training	Validation
1	Standard GS	Yield $(80\%)$	Yield $(20\%)$
2	Indirect GS	Height $(80\%)$	Height $(20\%)$ ^a^
3	Multi-trait indirect GS	Height $(80\%) +$ moisture $(80\%)$	Height $(20\%) +$ moisture $(20\%)$ ^b^
4	Multi-trait GS	Yield $(80\%) +$ height $(80\%)$	Yield $(20\%)$
5	Trait-assisted GS	Yield $(80\%) +$ height $(100\%)$	Yield $(20\%)$

^a^Prediction accuracies obtained as $r(\bar{x}_\mathrm{{Yield}},$GEBV$_\mathrm{{Height}})$

^b^GEBV$_\mathrm{{Height}}$ and GEBV$_\mathrm{{Moisture}}$ were scaled and weighted by their genetic correlations with $\bar{x}_\mathrm{{Yield}}$

In single-trait, multi-trait, and trait-assisted GS, genomic predictions of biomass yield itself were used to obtain *r*. In indirect GS, genomic predictions for a correlated trait (eg: height) were correlated with $\bar{x}$ of biomass yield to obtain *r*. In multi-trait indirect GS, genomic predictions for multiple correlated traits were scaled to have equal mean and variance before the following index was calculated:

5 $$\begin{aligned} \sum _{i=1}^{2}\mathrm{{cor}}_{g_{(Y,i)}}b_i, \end{aligned}$$

where $\mathrm{{cor}}_{g_{(Y,i)}}$ is the additive genetic correlation between trait *i* and biomass yield, and $b_i$ is the vector of GEBVs for trait *i*. Prediction accuracy of indirect multi-trait GS was calculated as correlation between this index and biomass $\bar{x}$. Multi-trait and trait-assisted GS differ only in that the latter uses $100\%$, rather than $80\%$, of correlated trait data for prediction of the focal trait. Thus, trait-assisted GS uses more total data points than multi-trait GS, including correlated trait phenotypes in the validation population. These strategies are similar to those used in Burgueño et al. (2012) for a multi-environment GS study. Analogously, predictions in multi-trait GS were entirely based on record of other lines, as in CV1. On the other hand, trait-assisted GS took advantage of correlated traits, similar to what was done in CV2 for correlated environments.

### Coincidence between models

Coincidence between $\bar{x}$ and GEBVs was calculated for the top and bottom $20\%$ individuals in each cross-validation run using the following coincidence index (CI) (Hamblin and Zimmermann 1986):

6 $$\begin{aligned} \mathrm{{CI}}=\frac{B-R}{T-R}, \end{aligned}$$

where *B* is the number of selected genotypes that is common in both models; *T* is the total number of selected genotypes; and *R* is the expected number of genotypes selected by chance. For example, repeated random selection of $20\%$ of genotypes (91 of 453) would yield an expected overlap of 18 genotypes ($20\%$ of 91) between random drawings.

All statistical analyses were conducted using R 3.0.3 R Core Team (2014) and the GBLUP model was fitted using the ASReml-R library (Butler et al. 2009). Phenotypic and genotypic information used, as well as scripts for all analysis performed in this paper can be found in https://github.com/samuelbfernandes/Trait-assisted-GS.

## Results

**Fig. 1:**
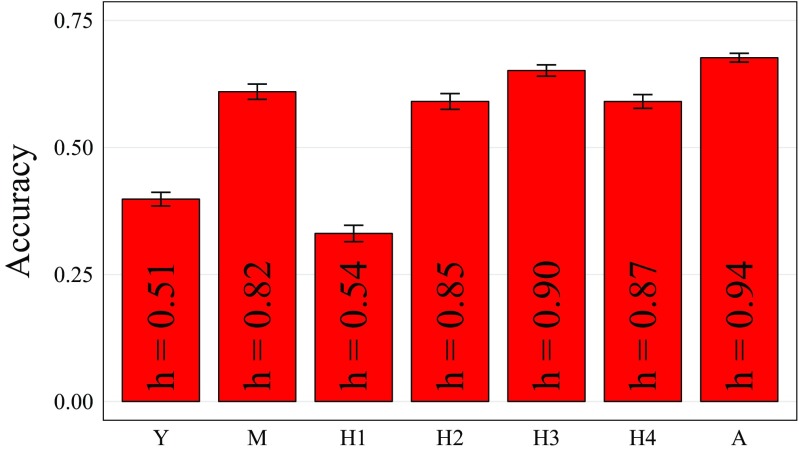
Prediction accuracy of standard GS for biomass (*Y*), moisture (*M*), height at 30 (*H*1), 60 (*H*2), 90 (*H*3), 120 (*H*4) DAP and the area under growth progress curve (*A*). Standard deviations across 30 cross-validation runs are shown. The square root of the heritability (*h*) is shown inside each bar

Prediction accuracy of the standard GS model was, in general, proportional to the square root of the genomic heritability for each trait (Fig. 1). The lowest accuracy in this study was obtained for *H*1 (0.33), followed by the one obtained for *Y* (0.40). On the other hand, the square root of the genomic heritability (*h*) for biomass (0.51) was slightly smaller than $h_{H1}$ (0.54). The highest *h* (0.94) and *r* (0.68) were obtained for *A*, with *H*3 close behind (Fig. 1). The other traits (*M*, *H*2 and *H*4) had similar *r* and *h*.

**Fig. 2:**
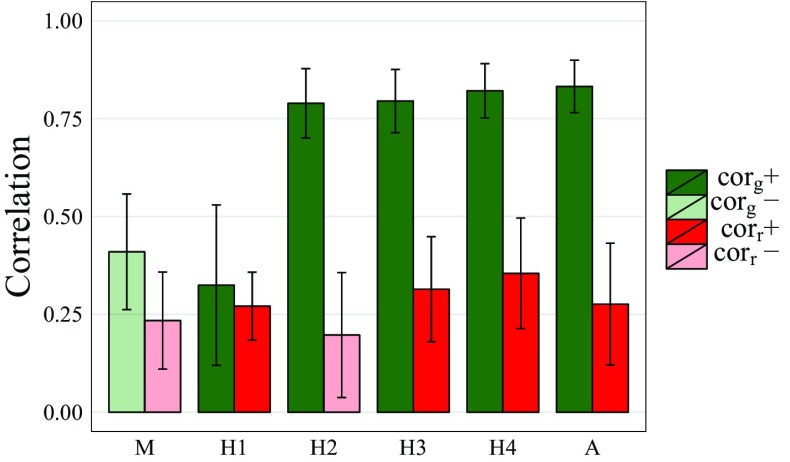
Genetic ($\mathrm{{cor}}_g$) and residual ($\mathrm{{cor}}_r$) correlations between biomass and moisture (*M*), height at 30 (*H*1), 60 (*H*2), 90 (*H*3) and 120 (*H*4) DAP. Positive ($+$) and negative (−) correlations are indicated by shading, and standard errors of correlations are shown

All traits were genetically correlated with biomass yield (Fig. 2). The genetic correlation between biomass yield and moisture was negative, whereas genetic correlations with plant height traits were all positive and increased with each successive plant height measurement. For *H*2, *H*3, *H*4 and *A*, genetic correlations with *Y* were greater than residual correlations with *Y*, suggesting that they could be useful for multi-trait prediction of *Y* (Schaeffer 1984).

**Fig. 3:**
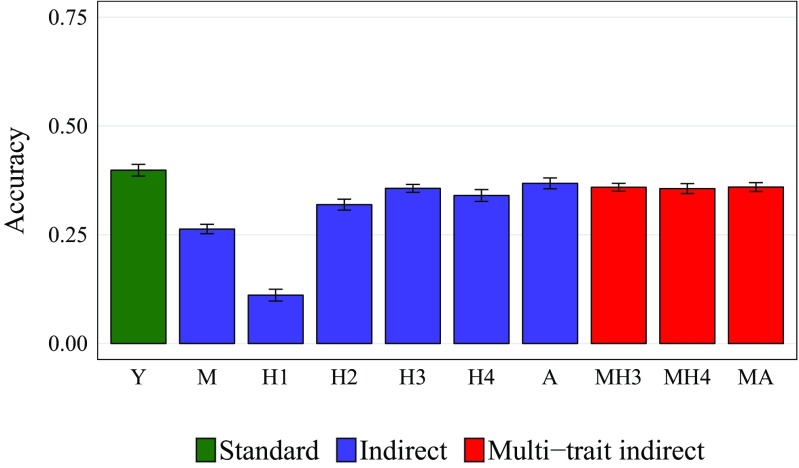
Prediction accuracy for biomass yield (*Y*) using indirect and multi-trait indirect GS with moisture (*M*), height at 30 (*H*1), 60 (*H*2), 90 (*H*3), 120 (*H*4) DAP and the area under growth progress curve (*A*) and combinations of these variables as correlated traits. Standard, direct GS is shown for comparison. Standard deviations across 30 cross-validation runs are shown

Prediction accuracies of indirect GS models (Fig. 3) were generally proportional to the genetic correlation of a correlated trait with biomass yield (Fig. 2). Prediction accuracy for *Y* using *H*3 data ($r_{Y/H3}$) was slightly higher than $r_{Y/H4}$ despite having a lower genetic correlation. The best prediction accuracy from indirect GS, $r_{Y/A}$, was nearly ($92.46\%$) as high as for standard GS. Multi-trait indirect GS did not show any advantage over single-trait indirect GS.

**Fig. 4:**
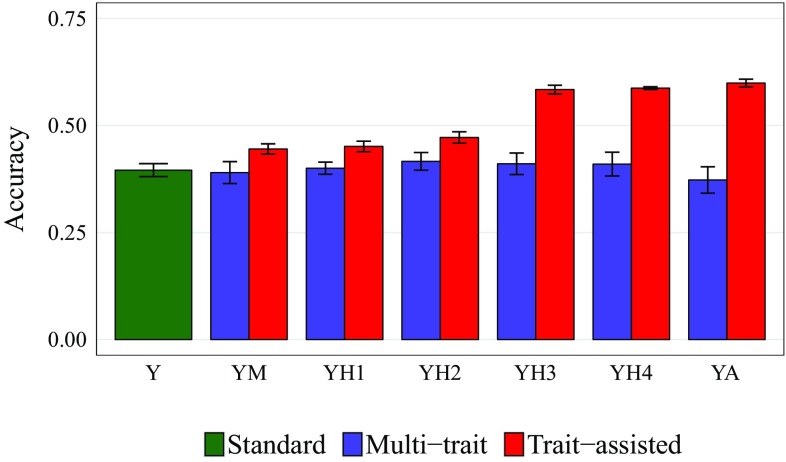
Prediction accuracy for biomass yield (*Y*) using multi-trait and trait-assisted GS with moisture (*M*), height at 30 (*H*1), 60 (*H*2), 90 (*H*3) and 120 (*H*4) DAP and the area under growth progress curve (*A*) as correlated traits. Standard, single-trait GS is shown for comparison. Standard deviations across 30 cross-validation runs are shown

Using information from correlated traits in the training population (multi-trait GS) did not provide any increase in prediction accuracy over the standard, single-trait GS model (Fig. 4). On the other hand, using information from correlated traits in both the training and validation populations (trait-assisted GS) increased prediction accuracy for biomass regardless of the secondary trait analyzed with *Y*, with the highest accuracy obtained for *YA* (0.60) (Fig. 4). Prediction accuracy increases with trait-assisted GS ranged from $11.8\%$ using *YM* to $50\%$ with *YA*, relative to standard single-trait GS. For highly correlated traits (*H*3, *H*4, and *A*), trait-assisted GS models maintained their advantage over standard GS even when the training population was reduced to $20\%$ of the dataset ($n= 90$), though this was not true for moderately correlated traits (*M*, *H*1, and *H*2; Fig. S1). Interestingly, the reduction in variance of GEBVs relative to $\bar{x}$ was also less dramatic for trait-assisted GS compared to the other GS models. Whereas, biomass yield $\bar{x}$ had a standard deviation of 2.13 tons/ha, single trait, multi-trait, and trait-assisted GEBVs had standard deviations of 0.85, 0.86, and 1.21 tons/ha respectively, using *A* as the correlated trait.

Coincidence indices (CIs) between the top and bottom $20\%$ of $\bar{x}$ and GEBVs were compared between single-trait, multi-trait, and trait-assisted GS models. In all cases CIs were below 0.5. However, CIs between trait-assisted GEBVs and $\bar{x}$ were higher than between single- and multi-trait GEBVs and $\bar{x}$ when the correlated trait was *H*2, *H*3, *H*4, or *A*. Higher CIs were observed for the bottom $20\%$ than for the top $20\%$, likely reflecting the asymmetric distribution of the underlying $\bar{x}$ (Table 2).

**Table 2:** Coincidence index between biomass $\bar{x}$ and GEBVs in multi-trait and trait-assisted GS models

Trait	Top $20\%$		Bottom $20\%$	
	Multi-trait	Trait-assisted	Multi-trait	Trait-assisted
*Y* ^a^	0.33 ± 0.02		0.34 ± 0.02	
*YM*	0.32 ± 0.02	0.35 ± 0.02	0.33 ± 0.02	0.37 ± 0.02
*YH*1	0.33 ± 0.02	0.36 ± 0.02	0.34 ± 0.02	0.35 ± 0.02
*YH*2	0.35 ± 0.02	0.40 ± 0.02	0.34 ± 0.02	0.40 ± 0.02
*YH*3	0.33 ± 0.02	0.40 ± 0.02	0.34 ± 0.02	0.44 ± 0.02
*YH*4	0.33 ± 0.02	0.39 ± 0.02	0.35 ± 0.02	0.44 ± 0.02
*YA*	0.30 ± 0.02	0.41 ± 0.02	0.35 ± 0.02	0.46 ± 0.02

Results are shown for a selection intensity of $20\%$ (top and bottom) with standard deviations

^a^Standard GS model is shown for comparison

We next compared the expected selection accuracy of multi-trait and trait-assisted GS to phenotypic selection and indirect phenotypic selection, given the heritabilities and genetic correlations observed for the focal trait (*Y*) and the correlated traits (*M*, *H*1, *H*2, *H*3, *H*4, *A*) in this study. Compared to phenotypic selection, multi-trait GS was always less accurate whereas trait-assisted GS was more accurate when using *H*3, *H*4 or *A* as correlated traits (Table 3). Compared to indirect phenotypic selection, both multi-trait and trait-assisted GS were less accurate when the correlated trait had a low genetic correlation with the focal trait (*M*, *H*1), and both were less accurate when this genetic correlation was high (*H*2, *H*3, *H*4, *A*).

**Table 3:** Expected selection accuracy of multi-trait and trait-assisted GS relative to phenotypic selection ($PS;\; r = h_Y$) and indirect phenotypic selection ($IPS;\; r = h_x*\mathrm{{cor}}_{g_{(x,Y)}}$), where *x* and *Y* are the correlated and focal traits

Traits	MTA/PS		MTA/IPS	
	Multi-trait	Trait-assisted	Multi-trait	Trait-assisted
*YM*	0.76	0.87	1.22	1.38
*YH*1	0.78	0.88	1.85	2.05
*YH*2	0.82	0.92	0.63	0.73
*YH*3	0.80	1.14	0.56	0.80
*YH*4	0.80	1.16	0.55	0.82
*YA*	0.73	1.18	0.47	0.76

## Discussion

In this study, we consider strategies for genomic selection of an expensive, low-heritability focal trait when correlated traits with higher heritability can be measured more easily, cost-effectively, or earlier in the life cycle. These strategies include single- and multi-trait direct and indirect GS, as well as a new approach we call trait-assisted GS.

### Single-trait GS

Marker-based prediction relies on good phenotyping, and prediction accuracy generally increases with heritability (Combs and Bernardo 2013). In this study, sorghum biomass yield showed low $h_\mathrm{{g}}^2$ (0.26) and moderate *r* (0.40). Similar results have been obtained in other crops such as wheat, where $h^2$ and *r* of biomass were 0.38 and 0.37, respectively (Combs and Bernardo 2013). In a study conduced by Lehermeier et al. (2014), *r* for biomass in corn varied from 0.17 in multi-parental to 0.41 in full-sib lines from a dent pool and from 0.30 in multi-parental to 0.48 in full-sib lines of a flint pool. GS offers the potential advantages of increasing selection intensity (Sonesson and Meuwissen 2009; Riedelsheimer et al. 2013) and allowing more selection cycles per unit time, both of which could result in higher genetic gain in comparison with phenotypic selection (Heffner et al. 2010). One previous study performed GS for biomass yield in sorghum (Yu et al. 2016), and found that *r* ranged from 0.69 using five-fold CV in a training set of 299 lines, to 0.76 in a validation set enriched for predicted-high and predicted-low lines, to 0.56 in an independent panel. The lower value of *r* in our study perhaps reflects the fact that our panel, while certainly not elite, had been pre-screened to exclude extremes of maturity variation, dwarfism, and lodging.

Height is usually a high-heritability trait (Heffner et al. 2011; Lipka et al. 2014; Burks et al. 2015), and the prediction accuracies of all height measurements except for the first one (*H*1, at 30 DAP) were higher then $r_Y$. Each height measurement was analyzed individually in addition to the area under growth progress curve (*A*). The *H*1 measurement by itself is clearly too early for accurate selection. Interestingly, *H*3 showed higher $h_\mathrm{{g}}^2$ and *r* than *H*4, possibly due to residual variation in maturity and lodging among genotypes that affected height measurements at the end of the season. The highest $h_\mathrm{{g}}^2$ and *r* were obtained for *A*. Given increasing adoption of high-throughput phenotyping techniques (Araus and Cairns 2014), more work could be done comparing the use of integrated measures such as *A* with multivariate models that include all individual time points.

### Indirect GS

Indirect GS using predictions of *H*2, *H*3, *H*4, or *A* to predict biomass appears promising, with the *A* model achieving $92.5\%$ of the prediction accuracy of the standard, direct GS model ($r_{Y/A}=0.37$; $r_Y=0.40$). Assuming that equivalent height heritabilities would be obtained from smaller plots, selection intensity and genetic gain could be increased by selecting on height instead of biomass in much larger population at equivalent field cost. An additional consideration in biomass sorghum is that measurement of vegetative biomass yield is incompatible with seed production. Indirect GS using an early-season trait such as *H*2 could potentially allow time for flowering induction and within-season seed production in selected lines, greatly reducing cycle length.

The failure of multi-trait indirect GS to increase prediction accuracy over single-trait indirect GS is very likely a consequence of the limited number of correlated traits measured in this study. Adding moisture information did not improve the ability of height models to predict biomass yield, but it seems likely that lodging, stand count, and a variety of architectural and spectral traits could be tested for improving multi-trait indirect GS models of biomass yield in sorghum.

### Multi-trait and trait-assisted GS

An alternative to indirect GS is to include one or more correlated traits along with the focal trait in a multi-trait model. In this strategy, marker effects for biomass yield are influenced by information from higher heritability traits [Mrode 2014, page 70] such as plant height. Multi-trait GS provided no advantage over standard, single-trait GS in this study, in contrast to several previous results using simulated (Guo et al. 2014; Calus and Veerkamp 2011) and real data (Jia and Jannink 2012; Schulthess et al. 2016), and in agreement with one previous study (dos Santos et al. 2016). Similar to what was obtained by Burgueño et al. (2012) in CV1, this result was somehow expected, since no information is recovered within lines across traits.

Trait-assisted GS is a new strategy in which correlated traits are used along with marker data in the validation panel. In the five-fold cross-validation scheme used in this study, this meant that $80\%$ of the yield data and $100\%$ of the height data were used, along with molecular markers, to predict the remaining $20\%$ of the yield data. Trait-assisted GS yielded dramatic improvements in prediction accuracy over all other GS models, with $r_{YA}$ showing an improvement of $50\%$ over prediction accuracy of *Y* in single-trait GS. Even $r_{MY}$ and $r_{H1Y}$ showed a $12\%$ improvement over the standard GS model, which was somewhat surprising given the relatively low genetic correlations of these traits with biomass (Schaeffer 1984; Galesloot et al. 2014). However, models including these traits did not maintain their advantage when the training population was reduced to a size as small as $20\%$ of the dataset (Fig. S1). These results suggest that even traits weakly correlated with a focal trait could be exploited in trait-assisted GS, given a training population of sufficient size.

Two other noteworthy results were obtained using the trait-assisted GS model. First, the standard deviations of the GEBVs were much higher in the trait-assisted models than in other GS models, though still greatly reduced relative to the standard deviations of $\bar{x}$. Second, the coincidence indices between biomass $\bar{x}$ and GEBVs were also highest for the trait-assisted GS models. These results suggest that differentiation of favorable and unfavorable genotypes is enhanced using trait-assisted GS, facilitating selection in a breeding program (Kadarmideen et al. 2003).

Trait-assisted GS has similarities with both multi-trait and indirect GS, as well as indirect phenotypic selection (IPS). Like IPS, selections are made using direct observation of correlated traits in individuals. Like standard GS, however, trait-assisted GS makes use of focal trait phenotypes in a training population, and genotypes in both training and selection populations, to perform selection. Like multi-trait GS, trait-assisted GS borrows information from correlated traits to inform focal trait marker effects. Trait-assisted GS shares all previously mentioned advantages of indirect (single- and multi-trait) GS for biomass sorghum improvement. However, it seems pointless to exclude focal trait data from a prediction model, as in canonical indirect GS and IPS, even if this data is limited in scope compared to the correlated trait data.

Several limitations of this study also deserve mention. First, Table 3 compares the expected selection accuracy of various strategies, but does not take into account possible differences in cycle length and selection intensity between them. Trait-assisted GS is probably intermediate to standard GS and traditional phenotypic selection in both cycle length and selection intensity. Second, this study used a highly structured pre-breeding population and no attempt was made to account for population structure. Therefore, we can expect that prediction accuracies of all GS models might be inflated relative to what might be observed in an elite population. Third, this study used $\bar{x}$ calculated across multiple years as input for the trait-assisted GS models. In an actual trait-assisted GS scenario in biomass sorghum, a single year of height data might be collected from a selection population, and used along with molecular markers and multiple years of height and yield data in a training population to perform selection.

Trait-assisted GS is probably intermediate to standard GS and traditional phenotypic selection in both cycle length and selection intensity. In biomass sorghum, for example, trait-assisted GS could reduce cycle length by selecting on correlated traits available prior to flowering (eg: *H*1, *H*2), and could increase selection intensity by reducing plot size for measurement of correlated traits with higher heritabilities (eg: one-row plots for plant height versus four-row plots for biomass yield).

## Conclusion

In this study, we show that phenotypic data on correlated traits in the validation set can be exploited to achieve substantial increases in prediction accuracy in a focal trait. This strategy should be useful whenever correlated traits can be measured earlier or more cheaply than a focal trait. Many plant and animal domesticates take years or decades to mature and allow full evaluation of yield and quality traits, and in these situations trait-assisted GS may allow dramatic increases in prediction accuracy and genetic gain.

### **Author contribution statement**

SBF and KOGD analyzed the data. DFF supported in the statistical analysis. SBF and PJB designed the field trials, collected the phenotypic data and wrote the manuscript. All authors read and approved the final manuscript.

## Electronic supplementary material

Below is the link to the electronic supplementary material.

Supplementary material 1 (PDF 95 kb)

## Abbreviations

NPGS: National plant germplasm system
GS: Genomic selection
Y: Biomass yield
M: Moisture
DAP: Days after planting
H1: Height at 30 DAP
H2: Height at 60 DAP
H3: Height at 90 DAP
H4: Height at 120 DAP
AIC: Akaike information criterion
GBLUP: Genomic best linear unbiased prediction
BLUP: Best linear unbiased prediction
A: Area under the growth progress curve
VCOV: Variance–covariance matrices
GEBV: Genomic estimated breeding value
IPS: Indirect phenotypic selection
MAF: Minor allele frequency
CI: Coincidence index
