# Supplementary material for: Efficiency of multi-trait, indirect, and trait-assisted genomic selection for improvement of biomass sorghum
Source: Theor Appl Genet. 2017 Dec 7;131(3):747–55. doi: 10.1007/s00122-017-3033-y (PMC5814553; doi:10.1007/s00122-017-3033-y)
Supplement: Supplementary file 1 — Supplementary material 1 (PDF 95 kb) [file 122_2017_3033_MOESM1_ESM.pdf]

Appendix

Table S1: Models and error variance-covariance structure (VCOV) used for analyzing Y, M, $H1$, $H2$, $H3$ and $H4$ in the first stage.

Year	Model	VCOV
2012	$Y_{ij} = \mu + G_i + B_j + e_{ij}$	$AR(1)\text{x}AR(1)$
2013	$Y_{ij} = \mu + G_i + B_j + e_{ij}$	I
2014	$Y_{ij} = \mu + G_i + B_j + e_{ij}$	$AR(1)\text{x}AR(1)$
2012	$M_{ij} = \mu + G_i + B_j + e_{ij}$	I
2013	$M_{ij} = \mu + G_i + B_j + e_{ij}$	I
2014	$M_{ij} = \mu + G_i + B_j + e_{ij}$	I
2012	$H1_{ij} = \mu + G_i + B_j + e_{ij}$	I
2013	$H1_{ij} = \mu + G_i + B_j + e_{ij}$	$AR(1)\text{x}AR(1)$
2014	$H1_{ij} = \mu + G_i + B_j + e_{ij}$	$AR(1)\text{x}AR(1)$
2012	$H2_{ij} = \mu + G_i + B_j + e_{ij}$	I
2013	$H2_{ij} = \mu + G_i + B_j + e_{ij}$	$AR(1)\text{x}AR(1)$
2014	$H2_{ij} = \mu + G_i + B_j + e_{ij}$	$AR(1)\text{x}AR(1)$
2012	$H3_{ij} = \mu + G_i + B_j + e_{ij}$	I
2013	$H3_{ij} = \mu + G_i + B_j + e_{ij}$	I
2014	$H3_{ij} = \mu + G_i + B_j + e_{ij}$	$AR(1)\text{x}AR(1)$
2012	$H4_{ij} = \mu + G_i + B_j + e_{ij}$	I
2013	$H4_{ij} = \mu + G_i + B_j + e_{ij}$	I
2014	$H4_{ij} = \mu + G_i + B_j + e_{ij}$	I

Table S2: Phenotypic correlation among the traits used in the study.

	Y	M	h1	h2	h3	h4	A
Y	1.00	-0.36	0.27	0.37	0.55	0.58	0.56
M		1.00	-0.03	-0.61	-0.45	-0.36	-0.51
h1			1.00	0.34	0.31	0.28	0.36
h2				1.00	0.66	0.59	0.80
h3					1.00	0.88	0.97
h4						1.00	0.92
A							1.00

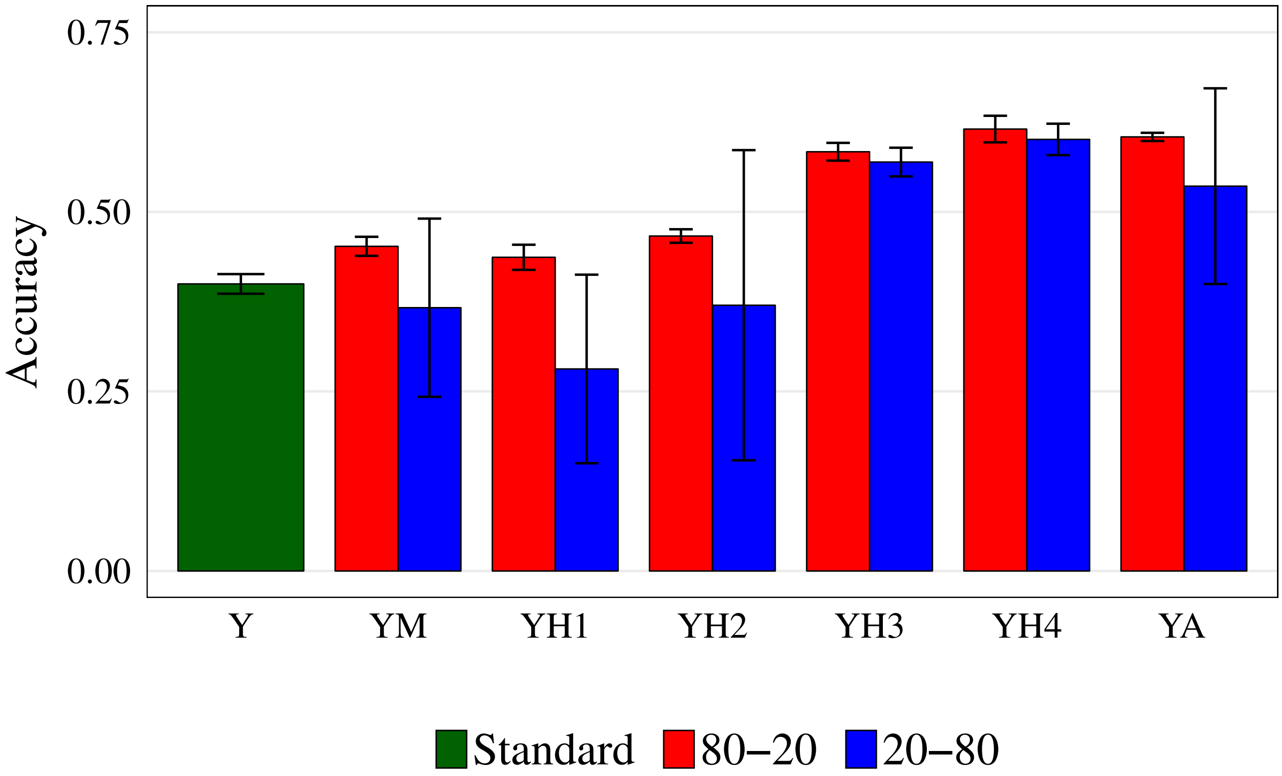

Fig. S1: Prediction accuracy for biomass yield (Y) using trait-assisted GS with moisture (M), height at 30 ($H1$), 60 ($H2$), 90 ($H3$) and 120 ($H4$) DAP and the area under growth progress curve (A) as correlated traits and comparing cross-validation using 80% ($80 - 20$) or 20% ($20 - 80$) of the lines as training population. Standard, single-trait GS is shown for comparison. Standard deviations across 30 cross-validation runs are shown.
